# Comparison of *Haemophilus parasuis* reference strains and field isolates by using random amplified polymorphic DNA and protein profiles

**DOI:** 10.1186/1471-2180-12-108

**Published:** 2012-06-15

**Authors:** Emilie S Zehr, Dennis V Lavrov, Louisa B Tabatabai

**Affiliations:** 1 Ruminant Diseases and Immunology, National Animal Disease Center, Agricultural Research Service, U. S. Department of Agriculture, Ames IA 50010, USA; 2 Department of Ecology, Evolution and Organismal Biology, Iowa State University, Ames, IA 50010, USA

**Keywords:** *Haemophilus parasuis*, RAPD, SDS-PAGE

## Abstract

**Background:**

*Haemophilus parasuis* is the causative agent of Glässer’s disease and is a pathogen of swine in high-health status herds. Reports on serotyping of field strains from outbreaks describe that approximately 30% of them are nontypeable and therefore cannot be traced. Molecular typing methods have been used as alternatives to serotyping. This study was done to compare random amplified polymorphic DNA (RAPD) profiles and whole cell protein (WCP) lysate profiles as methods for distinguishing *H. parasuis* reference strains and field isolates.

**Results:**

The DNA and WCP lysate profiles of 15 reference strains and 31 field isolates of *H. parasuis* were analyzed using the Dice and neighbor joining algorithms. The results revealed unique and reproducible DNA and protein profiles among the reference strains and field isolates studied. Simpson’s index of diversity showed significant discrimination between isolates when three 10mer primers were combined for the RAPD method and also when both the RAPD and WCP lysate typing methods were combined.

**Conclusions:**

The RAPD profiles seen among the reference strains and field isolates did not appear to change over time which may reflect a lack of DNA mutations in the genes of the samples. The recent field isolates had different WCP lysate profiles than the reference strains, possibly because the number of passages of the type strains may affect their protein expression.

## Background

*Haemophilus parasuis* causes Glässer’s disease in pigs, with symptoms of fibrinous polyserositis, pericarditis, polyarthritis, and meningitis [1]. *H. parasuis* also causes septicemia and pneumonia without polyserositis and can be isolated from nasal passages of healthy swine. Introduction of conventionally raised pigs into segregated early weaning herds may result in infection and high economic losses because the latter lack immunity to *H. parasuis*[2,3]. *H. parasuis* also remains a problem in many high health status herds. Economic losses in 2006 in the United States were estimated at $145 million dollars (Rodney B. Baker, Veterinary Diagnostic and Production Animal Medicine, Iowa State University, personal communication); [4].

*Haemophilus parasuis* strains are classified into 15 serovars based on immunodiffusion of heat-stable polysaccharide antigens [5,6]. However, reagents for serotyping field isolates are not readily available, and a large number of isolates cannot be identified by serotyping and are designated as nontypeable (NT) [7]. Other serotyping methods, such as the indirect hemagglutination test [7-9] have been employed to identify NT isolates. Nonetheless, there are still NT isolates that do not have serovar-specific reagents and cannot be characterized. The virulence of each serovar was determined in specific pathogen free pigs [5].

Molecular typing techniques are increasingly used to identify field isolates including NT isolates. These methods include polymerase chain reaction-restriction fragment length polymorphism (PCR-RFLP) [10,11], enterobacterial repetitive intergenic concensus-polymerase chain reaction (ERIC-PCR) [12,13], restriction endonuclease analysis [14,15], multilocus enzyme electrophoresis (MEE) [16], and multilocus sequence typing (MSLT) analysis [17]. The molecular typing methods have shown that considerable genetic diversity exists among strains of isolates of a particular serotype and that the genotyping techniques were more discriminating compared to conventional serotyping, especially for use in epidemiological studies. Each of these molecular typing techniques offers advantages and disadvantages.

For example, restriction endonuclease experiments [14,15] found distinct patterns of isolates from animals with systemic disease compared to respiratory isolates from healthy animals but restriction enzymes are expensive. The PCR-RFLP method uses restriction enzymes and sometimes does not generate multiple bands [11]. Multilocus sequence typing (MSLT) is a technique that studies housekeeping genes [17]. However, the latter procedure requires isolation of genomic DNA, performing PCR, and sequencing of PCR products. Both ERIC-PCR [12,13,18-20] and MSLT analysis [17] could detect strain variation but not all strains were classified as virulent or avirulent.

Although ERIC-PCR has recently been extensively used to study the epidemiology of *H. parasuis* isolates [19-21], the random amplified polymorphic DNA (RAPD) technique has not been utilized for this purpose. However, RAPD has been used to distinguish other gamma-proteobacteria, including *Salmonella* spp. [22], *E. coli* O157 [23], and *Klebsiella pneumonia*[24]. Both ERIC-PCR and random amplified polymorphic DNA (RAPD) are global techniques since known primers can be easily synthesized, reagents are affordable and readily obtained, and the techniques have high levels of reproducibility.

In the PCR-based RAPD method, DNA does not have to be double-stranded, highly purified, or of high molecular weight [25]. Both ERIC-PCR and RAPD can utilize DNA from crude lysates [13,26,27] which shortens the time needed for completing the assays. However, most laboratories prefer to purify genomic DNA before it is used in genome-based techniques, especially if it is to also be used in assays other than the ERIC-PCR technique [16,18-21,28].

The ERIC-PCR technique uses higher annealing temperatures (approximately 50–58°C) and longer primers (20 nucleotides) than the RAPD method. These primers are specific for areas of the genome that are highly conserved and include an inverted repeat. The RAPD assay uses low stringency conditions of approximately 30–36°C annealing temperatures and short (10 nucleotide) primers. One or more of these arbitrarily chosen RAPD primers can anneal at multiple locations throughout the genome and amplify many products of the template DNA.

In addition to genomic-based methods, protein-based methods offer a different and complementary approach. Whole cell protein (WCP; [29-32] profiles or outer membrane protein profiles [33] of *H. parasuis*, which use a sodium dodecyl sulfate polyacrylamide gel electrophoresis (SDS-PAGE) technique have been described. These studies suggested that isolates from systemic sites had unique protein profiles. Isolates from respiratory sites had different protein profiles than the systemic isolates had. The 36–38.5 kDa proteins were described as virulence markers based on the isolation site of the strain [32].

This work analyzed the DNA and protein profiles of 46 *H. parasuis* reference and field isolates. Random amplified polymorphic DNA is a molecular typing technique that is often used to differentiate closely related strains. It is especially sensitive to strain variation when three optimized primers are employed [34-36]. Random amplified polymorphic DNA may detect single base changes in genomic DNA and genetic maps consisting of RAPD markers can be generated more efficiently than by using RFLP targeted PCR-based methods [28]. Intra-specific variation in the RAPD patterns can be observed for each primer and the sequence complexity of small plasmids is unlikely to contribute to the patterns [26]. However, bacteriophage and larger plasmids with transposons could possibly mediate horizontal gene transfer between strains and increase RAPD heterogeneity [18]. By using the relatively simple and economical RAPD technique, known primer sequences can be utilized by different laboratories, making it a standardized technique and amenable to epidemiological studies. However, interpretation of gel electrophoresis results could introduce some variability between laboratories. The objectives of this study were to compare the relatedness of the reference strains and field isolates based on the RAPD and WCP lysate profiles and to determine if clustering that occurred was related to the site of isolation or to the pathogenicity of the strain.

## Results

### Comparison of RAPD profiles and pattern analysis

**Figure 1:**
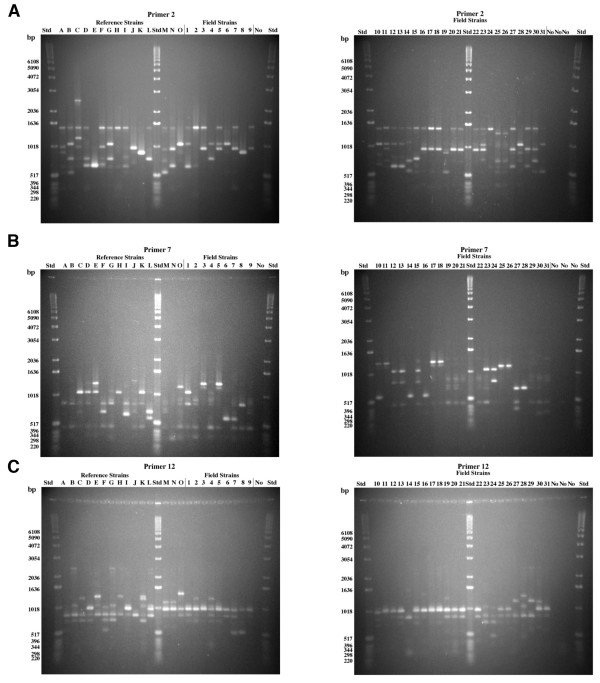
**RAPD analysis of *H. parasuis *strains using primer 2 (panel A), primer 7 (panel B), and primer 12 (panel C).** Reference strains A-O are described in Table 1. Reference strains were obtained between 1978 and 1990. Field strains 1–31 are described in Table 2. Field strains 1–24, 25–29, 30–31 were obtained in 2004, 1999, and 1984, respectively. Each lane was loaded with 10 μl of PCR amplification product containing approximately 30 ng of DNA. A DNA control (no cells) was included in lanes marked “No”. The Standard (Std) was a 1 kb DNA ladder.

**Figure 2:**
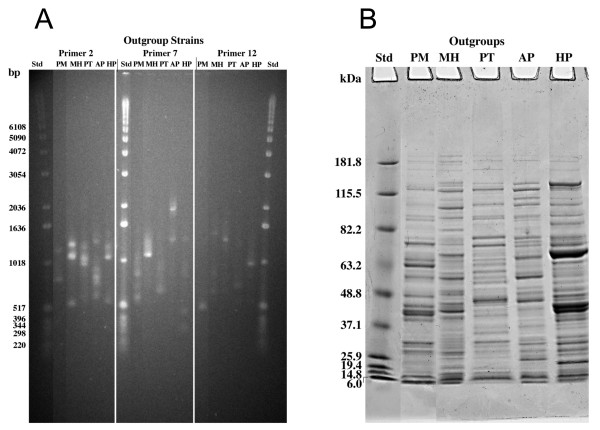
**Characterization of outgroup strains by RAPD analysis and by WCP profiles.** (**A**) RAPD analysis of four outgroup strains using primers 2, 7, and 12: *Pasteurella multocida* (PM), *Mannheimia haemolytica* (MH), *Pasteurella trehalosi* (PT) and *Actinobacillus pleuropneumoniae* (AP). The *H. parasuis* IA84-29755 (HP) fingerprint is shown for comparison to outgroup strains. Std was 1 kb DNA ladder; (**B**) SDS-PAGE analysis of WCP lysates of outgroup strains, samples were identical to those in panel A; Molecular weights (Std) are indicated in kilodaltons.

Of the three primers used for genotyping, primer 2 had an intermediate number of bands; primer 7 had the most polymorphic DNA bands; and primer 12 had the least number of polymorphic DNA bands (Figure 1). Identical patterns were obtained for each isolate for each primer when the assays were performed in triplicate. Band sizes of DNA ranged between 220–3054 base pairs (bp). There were bands that were more densely stained than others, but all bands were treated identically. Four outgroup strains that were in the same family as *H. parasuis* but from different genera were included in the analysis. Fingerprints of DNA were unique for each outgroup isolate and different from the fingerprint of *H. parasuis* for each primer (Figure 2A).

**Table 1:** **Description of *H. parasuis *** **reference strains**^**a**^

**#**	**Serovar**	**Strain**	**Country**	**Isolation Site**	**Diagnosis**	**Virulence**^**b**^
A	1	No. 4	Japan	Nose	Healthy	H
B	2	SW140	Japan	Nose	Healthy	L+
C	3	SW114	Japan	Nose	Healthy	A
D	4	SW124	Japan	Nose	Healthy	L+
E	5	Nagasaki	Japan	Meninges	Meningitis,	H
					septicemia	
F	6	131	Switzerland	Nose	Healthy	A
G	7	174	Switzerland	Nose	Healthy	A
H	8	C5	Sweden	Unknown	Unknown	L-
I	9	D74	Sweden	Unknown	Unknown	A
J	10	H367^c^	Germany	Unknown	Unknown	H
K	11	H465	Germany	Trachea	Pneumonia	A
L	12	H425	Germany	Lung	Polyserositis	H
M	13	84-17975	United States	Lung	Unknown	H
N	14	84-22113	United States	Joint	Septicemia	H
O	15	84-15995	United States	Lung	Pneumonia	L+

^a^originally published by Kielstein and Rapp-Gabrielson (1992) and adapted by Zehr and Tabatabai (2011).

^b^H, Highly virulent, death of pig within 96 h post-inoculation; L+, Polyserositis and arthritis at necropsy; L-, Mild clinical symptoms; A, Avirulent, no clinical symptoms at necropsy as described by Kielstein and Rapp-Gabrielson (1992).

^c^H367 (serovar 10) is a field strain with the same characteristics as the original H555. Reference strain H555 was lost during culture passage prior to our acquisition of the reference strains above.

**Table 2:** **Description of *H. parasuis *** **field isolates**^**a**^

**#**	**Serovar**	**Strain**	**U.S. state of origin**	**Isolation site**	**Diagnosis**
1	Unk^b^	10680	Oklahoma	Lung, heart	Septicemia
2	Unk	12939	North Carolina	Lung, heart	Pneumonia, polyserositis
3	Unk	15677	Minnesota	Brain, heart	Pneumonia, polyserositis
4	Unk	17321	Illinois	Brain, lungs	Septicemia, pneumonia, meningitis
5	Unk	24054	Iowa	Lung	Septicemia, polyserositis
6	4	24996	Iowa	Lung	Pleuritis, septicemia
7	12	25718	Iowa	Heart	Pericarditis
8	Unk	28803	Iowa	Cerebrospinal fluid, lung	Meningitis, septicemia
9	13	29612	North Carolina^c^	Brain, Lung, Joint	Pneumonia, meningitis
10	4	29613	North Carolina^c^	Lung	Septicemia
11	5	29614	North Carolina^c^	Multiple sites	Septicemia
12	Unk	29814	Iowa	Lung, joint	Septicemia
13	Unk	29864	Iowa	Brain, joint	Meningitis, septicemia
14	5	30059	Iowa	Lung	Septicemia
15	Unk	32585	Iowa	Lung	Pneumonia, polyserositis
16	12	33105	Missouri	Lung	Meningitis, pneumonia, polyserositis, septicemia
17	12	33206	Iowa	Brain, lung, joint	Pneumonia, septicemia
18	12	33808	Minnesota	Lung, joint	Polyserositis, septicemia
19	5	34086a	Iowa^d^	Lung, joint	Septicemia
20	5	34086b	Iowa^d^	Lung, joint	Septicemia
21	4	34086c	Iowa^d^	Lung, joint	Serositis, septicemia
22	4	34086d	Iowa^d^	Lung, joint	Pneumonia, pleuritis, septicemia
23	2	35036	Iowa	Lung	Pneumonia, polyserositis
24	Unk	1269	Iowa	Heart, lung	Septicemia
25	Unk	831541^e^	Iowa	Probably lung	Unknown^f^
26	Unk	831542^e^	Iowa	Probably lung	Unknown^f^
27	Unk	464-99^e^	North Carolina	Joint	Polyserositis
28	Unk	685-99^e^	North Carolina	Pericardial sac	Polyserositis
29	Unk	1050-99^e^	North Carolina	Lung	Polyserositis
30	Unk^b,g^	2170B	Iowa	Joint	Polyserositis
31	5	84-29755	Iowa	Lung	Pneumonia, pleuritis

^a^originally published by Zehr and Tabatabai (2011).

^b^Unk, did not serotype to serovars 2, 4, 5, 12, 13, or 14.

^c^Isolated from same farm site.

^d^Isolated from same animal.

^e^”Old” field strains obtained in 1999.

^f^Sick animal.

^g^Originally serotype 4 in 1984.

**Figure 3:**
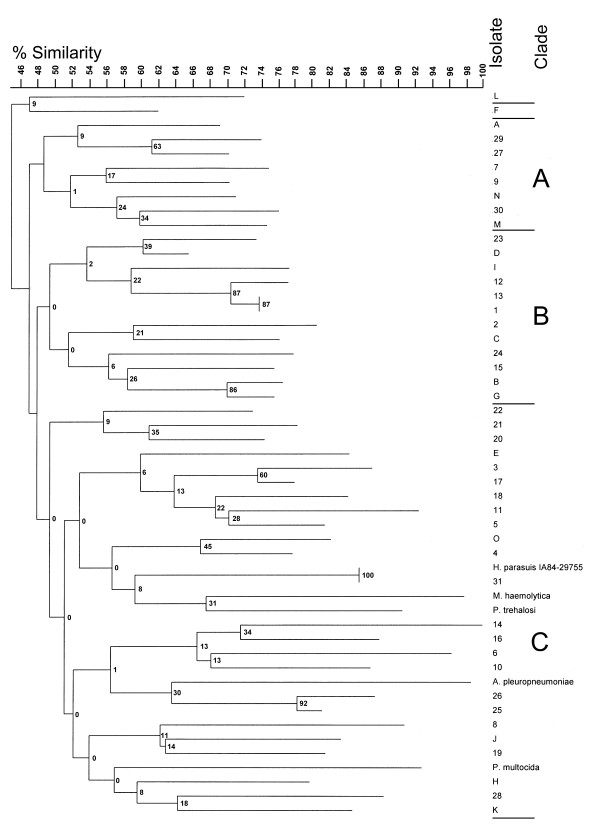
**Dendrogram grouping based on the composite RAPD electrophoretic band patterns of representative *H. parasuis *strains and outgroup strains.** Band patterns from all three single-primer experiments were combined to obtain a composite-primer RAPD dendrogram. Reference strains are designated A-O (Table 1), field isolates are designated 1–31 (Table 2), and outgroups are *Pasteurella multocida* (PM), *Mannheimia haemolytica* (MH), *Pasteurella trehalosi* (PT) and *Actinobacillus pleuropneumoniae* (AP). Three clade designations are shown. Reference strains were obtained between 1978 and 1990. Field strains 1–24, 25–29, 30–31 were obtained in 2004, 1999, and 1984, respectively. Numbers at the nodes indicate percentages of bootstrap values after 1000 replicates.

A composite dendrogram prepared from the RAPD data obtained by using three primers is shown in Figure 3. At 48.5% similarity, this dendrogram showed three clades (A, B, and C) and two unique reference serovars, one virulent isolate (L) from Germany which caused polyserositis and one avirulent nasal isolate (F) from Switzerland from a healthy animal. All except one of the isolates in Clade A were systemic. Clade B contained three avirulent reference strains from Sweden (I), Japan (C), and Switzerland (G). The remaining isolates in Clade B were isolated from “healthy” animals but were virulent (B and D) reference strains or systemic (1–2, 12–13, 15, 23–24) field isolates. Clade C isolates were systemic *H. parasuis* with the exception of one avirulent reference strain isolated from the trachea of a pig diagnosed with pneumonia from Germany (Figure 1, strain K) as well as all outgroup strains, *M. haemolytica, P. trehalosi, A. pleuropneumoniae, and P. multocida.* Duplicate cultures of *H. parasuis* IA84-29755 (systemic field isolate 31) and two outgroup strains (*M. haemolytica* and *P. trehalosi*), which are closely related phylogenetically, were grouped within one branch of Clade C. Duplicate cultures of *H*. *parasuis* field isolate 31 (IA84-29755) were included as controls and gave identical fingerprints when analyzed with primers 2, 7 and 12.

Both of the recent field isolates in Clade A (7 and 9) could be serotyped and 79% of the recent field isolates in Clade C (6, 14, 10–11, 16–22) were typeable, whereas 72% of the recent field isolates (1–2, 12–13, 15, 24) in Clade B were classified as “Unk”. Three isolates (20–22) from the same animal but with two different serotypes (4 and 5) clustered in the same clonal grouping (Figure 3).

### Comparison of SDS-PAGE protein profiles and pattern analysis

**Figure 4:**
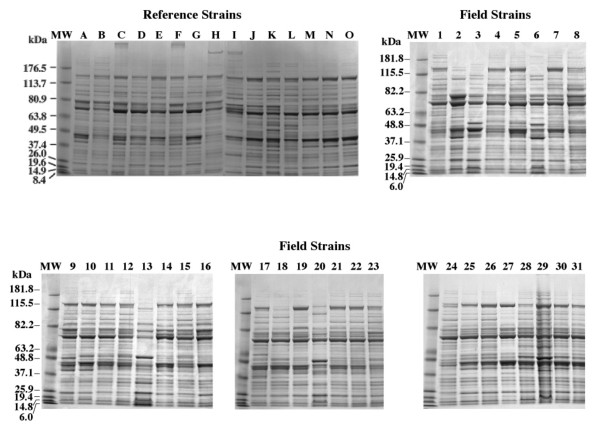
**SDS-PAGE profiles of representative *H. parasuis *strains. Gradient SDS-PAGE gels of WCP lysates were stained with Coomassie Brilliant Blue R250.** Reference strains A-O are described in Table 1. Reference strains were obtained between 1978 and 1990. Field strains 1–31 are described in Table 2. Field strains 1–24, 25–29, 30–31 were obtained in 2004, 1999, and 1984, respectively. Each lane was loaded with 10 μg of protein. Molecular weights (MW) are indicated in kilodaltons.

Protein bands between 8 and 180 kilodalton (kDa) were present in all of the reference strains and field isolates (Figure 4), as well as a few bands higher than 180 kDa in four of the reference strains C, F, H, and I, respectively. The latter reference strains corresponded to serovars 3, 6, 8, and 9, respectively, which all designated as avirulent. Isolates gave identical patterns when the analysis was performed in triplicate. Each serovar showed unique band patterns, but there were also common protein bands among the reference serovars (lanes A-O) and field isolates (lanes 1–31). For example, reference strains C and F showed a common protein at 253 kDa; and reference strains H and I showed a common band at 217 kDa. All reference strains (lanes A-O) and field isolates 25–31 expressed prominent bands at 140 kDa and 70 kDa and all strains except reference strains B and H (serovars 2 and 8, respectively) showed prominent bands at approximately 40 kDa. Visual inspection of the protein profiles of the field strains 25–31 (Figure 4) showed that these were similar to but not identical to reference strains K and L. Field strains 1–24 protein profiles were more heterogeneous than the reference strains or field isolates 25–31 protein profiles. Field isolates 3, 6, 13, 20, and 29 all had major protein bands at approximately 50 kDa, which were not apparent in the other protein profiles. Outgroup strains (Figure 2B) had unique WCP lysate patterns, which differed from the *H. parasuis* pattern, on an SDS-PAGE gel.

**Figure 5:**
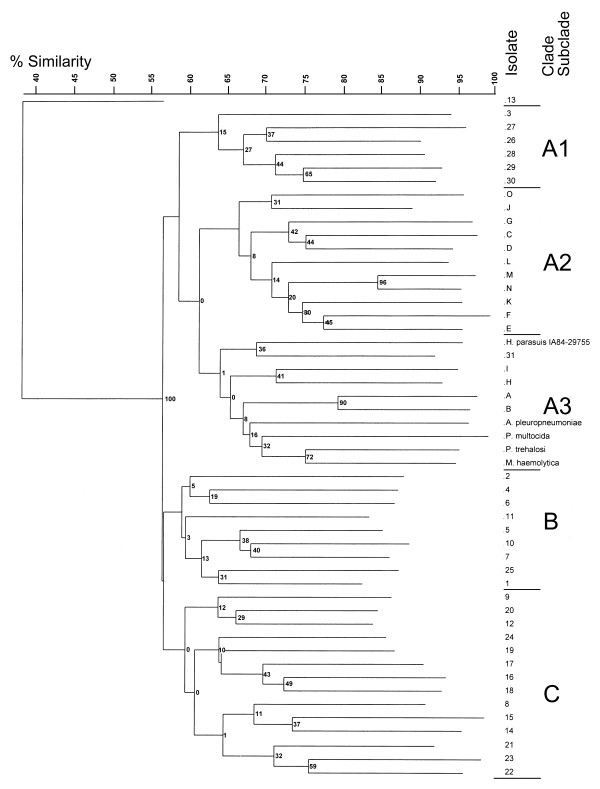
**Dendrogram grouping based on the SDS-PAGE WCP lysate profiles.** Reference strains are designated A-O (Table 1), field isolates are designated 1–31 (Table 2), and outgroups are *Pasteurella multocida* (PM), *Mannheimia haemolytica* (MH), *Pasteurella trehalosi* (PT) and *Actinobacillus pleuropneumoniae* (AP). Reference strains were obtained between 1978 and 1990. Field strains 1–24, 25–29, 30–31 were obtained in 2004, 1999, and 1984, respectively. Three clade and three subclade designations are shown. Numbers at the nodes indicate percentages of bootstrap values after 1000 replicates.

The neighbor joining dendrogram showing phylogenetic analysis of WCP lysates (Figure 5) used a band optimization of 1.12% and a band position tolerance of 1.1% and had one unique isolate (field strain 13 which was isolated from the brain and joint and had the 50 kDa band). Three clades (A, B, and C) at 58.5% similarity were generated and three subclades of Clade A at 63% similarity were produced. Subclade A1 contained all systemic field isolates (Figure 5, Table 2). Subclade A2 contained eleven of the fifteen original reference strains of various pathogenicities and isolation sites (Table 1). Subclade A3 contained four of the fifteen original reference strains of varied diagnosis as well as the duplicate systemic field strains *H. parasuis* (field isolate 31 and IA84-29755) and all of the outgroup strains. Clade B contained field isolate 25 from 1999 and eight systemic field isolates (1–2, 4–5, 6–7, 10–11) from 2004 and Clade C contained 14 systemic field isolates (8–9, 12, 14–24) from 2004 (Figure 5, Table 2).

Isolates in Clades B and C clustered all of the systemic type and Subclade A2 strains were entirely of the reference type, including four (C, F, G, K) of the five avirulent strains. The majority (four out of five) of field isolates from 1999 (26–29) were clustered in Subclade A1 (Figure 5). Additionally, all three of the North Carolina isolates (27–29) grouped in Subclade A1. There appeared to be some discrimination as to state of origin between isolates in Clades B and C because there were three North Carolina (2, 10–11), one Illinois (4), and one Oklahoma (1) isolates among the nine Clade B isolates whereas there were only one North Carolina (9), one Missouri (16), and one Minnesota (18) isolates among fifteen Clade C isolates. As with the RAPD neighbor joining analysis (Figure 3), recent field isolates seemed to group by serotype with 56% and 27% of the isolates in Clades B and C, respectively, not being serotyped to serovars 2, 4, 5, 12, 13, or 14.

### Discrimination of isolates using Simpson’s index of diversity

Simpson’s index of diversity estimates the discriminatory ability of typing systems by calculating the discrimination index (D). D is the probability that two unrelated strains randomly selected from the test population are in two different typing groups. The only RAPD with a single primer that gave a significant index level of discrimination above 90% was RAPD7 (Table 3). Groups and singletons were determined by using 55% similarity for the composite RAPD (Figure 3) and 63% similarity for the WCP lysate (Figure 5). Combining the results of all 3 primers gave an index of 94.11%. While the WCP lysate index was less than 90%, combining it with the composite RAPD gave an index of diversity of 97.3%.

**Table 3:** **Discrimination of isolates based on characterization method**^**a**^

**Characterization method**	**No. of groups**	**Simpson’s index of diversity**	**95% confidence level**	**No. of samples in largest group**
RAPD2^b^	17	85.70	78.44-92.96	15
RAPD7^c^	18	92.17	88.59-95.76	8
RAPD12^d^	19	89.66	84.43-94.90	11
RAPDC^e^	16	94.11	92.07-96.15	6
WCP^f^	9	88.60	84.77-92.43	11
WCP/RAPDC^g^	63	97.30	96.63-97.97	15

^a^Results of various characterization methods with the respective Simpson’s index of diversity value, 95% confidence interval, and the number of samples in the largest group produced by the method.

^b^RAPD bands using only primer 2.

^c^RAPD bands using only primer 7.

^d^RAPD bands using only primer 12.

^e^Composite RAPD combining bands from all three primers.

^f^Whole cell protein lysate.

^g^Combination bands from whole cell protein lysate and composite RAPD.

## Discussion

This study was undertaken to utilize the RAPD technique and SDS-PAGE protein profiles in order to compare 15 reference strains and 31 field isolates of *H. parasuis* to establish if a relationship existed between a particular clustering profile or if there was a relationship to the site of isolation or to the pathogenicity of the strain. The clinical origin and pathogenesis of a strain is an indication of its virulence, but conclusions as to its virulence cannot be made in our study because pathogenesis studies were not conducted in specific pathogen free pigs [5]. However, the virulence potential of *H. parasuis* strains, based on their serotype classification, isolation sites and the presence or absence of major proteins with molecular weights between 36 and 38.5 kDa, has been investigated [30,33,37]. Some of the expressed proteins in our recent field isolates may be called virulence markers but no direct association of the 40 kDa proteins could be made. Few laboratories have the ability to serotype *H. parasuis* isolates because of the lack of reagents. Therefore, a genome-based method and a phenotypic analysis of the reference strains and field isolates were emphasized in our study.

Neighbor joining analysis based on Dice coefficients of similarity was used to compare RAPD and protein (WCP lysate) profiles of the reference strains and field isolates. The samples were statistically discriminated better by using the composite RAPD technique than by using the WCP lysate technique but combining the results of both techniques gave a high discrimination index. Outgroups were included to compare the presence or absence of bands in these isolates to the bands in the more closely related *H. parasuis* isolates. The only monophyletic ingroup with the four “outgroups” was the SDS-PAGE dendrogram as determined by the neighbor joining analysis (Figure 5, Clade A3). The results suggest that the four outgroup species selected may have been too closely related to *H. parasuis* to act as a true outgroup. Dijkman et al. [20] were also unable to discriminate *A. minor* and *A. porcinus* strains from *H. parasuis* strains in an ERIC-PCR technique. Additionally, Olvera et al. [18] could not demonstrate that *A. indolicus* and *A. minor* strains were outgroups to *H. parasuis* strains when they used the variation of the partial *hsp60* sequence of *H. parasuis* as a classification tool.

Others have shown that the geographic distribution or age of the isolate may cause the “outgroup” to act as an ingroup [38] and that if the isolates in the study were too closely related, then the outgroups could be rerooted to locations within phylogenetic trees [39]. A fourth possibility for the lack of outgroup observance in the dendrograms could be that horizontal gene transfer has occurred between the outgroup species and *H. parasuis*[40], which would cause unexpected similarities and unusual phyletic patterns [18]. This theory is supported by the presence of bacteriophages in *H. parasuis*[41-43], *E. coli*[44], *P. multocida*[45], *M. haemolytica*[46], and *P. trehalosi*[47], plasmids in *H. parasuis*[48] and *A. pleuropneumoniae*[49], and a DNA uptake sequence in *H. parasuis*[50].

Although isolates from known systemic sites [51] (lung in an animal with polyserositis, joint, brain, heart, or septicemia) were able to be separated into groups by the RAPD technique described here, the composite diagram of the three individual primers ultimately showed a limited degree of relatedness based on pathogenicity among the reference strains and the 31 field strains. The strains showed high heterogeneity with the RAPD method which indicated possible horizontal transfer of genes or chromosomal recombination between unrelated and potentially transient strains.

Wang et al. [25] compared RAPD and MEE and found that RAPD data that combined five primers was more discriminatory than MEE tests that used 34 enzymes. The ERIC-PCR technique is a comparable method to RAPD. Zulkifli et al. [52] found RAPD to be more discriminatory than ERIC-PCR. Some *H. parasuis* isolates were not able to be assayed by using the ERIC-PCR [20] because they gave no or very poor results. Recent studies have found a high diversity of *H. parasuis* strains isolated in various geographic areas but have not been able to assign a clear correlation between virulence or serovar and ERIC-PCR clusters [19-21]. This conclusion agrees with other *H. parasuis* ERIC-PCR studies [12,18]. Macedo et al. [21] reported that the highest diversity was in the NT isolates which discriminated 23 genotypes. Our RAPD dendrogram also indicated high diversity of the *H. parasuis* strains, with only field isolates 1 and 13 being identical. Although there was no definite correlation between serovar and pathogenicity, most of the isolates that were serotypeable and from diseased animals clustered in Clade C.

Other genomic methods such as MEE and MLST [16,17], also did not completely discriminate field isolates of *H. parasuis*. Blackall et al. [16] found 34 different electrophoretic types from 40 field isolates and 8 reference serovars, which clustered into 2 major subdivisions, which were not associated with virulence. Olvera et al. [17] concluded that subgroups of 120 field isolates and 11 reference serovars clustered into branches containing avirulent, nasal isolates and virulent, systemic isolates. However, 36 additional clinical isolates did not cluster within the virulent branch.

Two different studies [53,54] combined serotyping and IHA methods and concluded that isolates of serovars 4, 5, 13, and NT isolates were the most prevalent in 2004 and 2005, with serovar 4 the most frequently isolated from the respiratory tract while NT isolates were usually systemic isolates. This study’s field isolates were known to be systemic except for isolates 25 and 26, and included serovars 2, 4, 5, 12, and 13, identified by available serotyping reagents. The serovars used in this study were the six most prevalent in the United States and Canada [51,55]. The range of NT (15-31%) to the frequency of identification of serovars 2, 4, 5, 12, 13, and 14 (76-41%), respectively, by immunodiffusion [32] compares to the frequencies of our “Unk” (51.6%) and six identified serovars (48.3%). Some of our field isolates may have lost the expression of their polysaccharide capsule *in vitro* and may not be able to be serotyped presently [12,51] as can be inferred from field isolate 30, which was serotype 4 in 1999 but “Unk” in our study. Field isolate 30 may have lost an enzyme involved in the polysaccharide capsule synthesis. All of our field isolates of known serotype were associated with animals with systemic disease. The majority of field isolates of known serotype were in clade C of the RAPD experiment except for isolates 7, 9, and 23 and in clades B and C of the WCL experiment. Rapp-Gabrielson and Gabrielson [51] and Olvera et al. [17] noted that the distribution of *H. parasuis* serovars isolated from healthy animals may differ from that found in diseased animals and that more than one serovar could be isolated from the same animal or same isolation site. Our study also identified isolates with different serovars within the same farm site (field isolates 9–11) and in from the same isolation sites in the same animal (field isolates 19–22). “Unk” isolates with septicemia were the most prevalent, indicating that NT isolates harboring bacteriophages, plasmids, or repetitive elements may influence genotyping methods [40]. There may not have been a correlation between serotype and RAPD because only a small number of genes is involved in serotyping while the entire genome is analyzed with the RAPD technique [22].

Our SDS-PAGE results agree with those of Oliviera and Pijoan [30] who reported that isolates from systemic sites were usually virulent and clustered together as shown by using a computer-based analysis of protein profiles from serovars 1, 2, 4, 5, 7, 12, 13, 14 and nontypeable (NT) isolates. Their results are similar to protein profiles described in our study for field isolates and their isolation sites and pathogenesis as shown in the WCP lysate dendrogram of Figure 5 and Table 2. The field strains clustered in Subclade A1 and Clades B and C were primarily systemic. Ruiz et al. [33] found different OMP profiles between isolates from healthy pigs and those from diseased pigs. However, they concluded that respiratory isolates were more heterogeneous than systemic isolates.

Four studies have stated that a protein of approximately 36–38.5 kDa may be associated with Glässer’s disease [29,30,33,56]. In this work, a protein band was observed at approximately 40 kDa in all of the field isolates and thirteen of fifteen of the reference strains (Figure 4). The results shown for the WCP lysate dendrogram (Figure 5) imply that protein expression may be related to age or number of passages of the isolate*in vitro*, because reference strains clustered together, as did the “old” field strains (26–29) isolated in 1999 (Figure 5, Subclades A2 (C-G, J-O), A3 (A-B, H-I), and A1 (26–29), respectively). The phenotypic change of an isolate after serial passage was also reported by Rapp-Gabrielson and Gabrielson and Oliviera et al. [12,57]. Although we had only seven samples from North Carolina, three isolates (27–29) from 1999 grouped together in Subclade A1 of the SDS-PAGE neighbor joining dendrogram (Figure 5). Our WCP lysate patterns easily discriminated between *A. pleuropneumoniae* serotype 1 and *H. parasuis* as well as the other three outgroup strains (Figure 2B). Identical *H. parasuis* field isolates (*H. parasuis* IA84-29755 and 31) (Figure 5), bands did not match sufficiently to obtain identity in the protein profile computer analysis. This may have been because the bands were not fully “matched” in the Gel Compar II program. They were, however, in the same clonal branch of Subclade A3.

Oliviera and Pijoan [30], Kielstein and Rapp-Gabrielson [5], Rosner et al. [58] and Blackall et al. [59] did not find any correlation between virulence and serotype of the isolate. However, the results reported in this study seem to indicate an association of virulence with isolates of Clade C in the WCP lysate analysis. There also seemed to be more serotypeable isolates among the recent field isolates of Clade C. This work also found that the genome-based RAPD composite analysis method (Figure 3) showed more heterogeneity than the protein-based WCP lysate analysis, which grouped isolates according to length of passage *in vitro* and to possible geographic origin (Figure 5). The underlying genome did not change as much as the protein expression did over time [10].

The recent field isolates from this study were obtained from swine diagnosed mostly with septicemia caused by serovars 2, 4, 5, 12, and 13. All of the isolates from diseased animals grouped into clades in the RAPD neighbor joining dendrogram containing systemic isolates (Figure 3, Clades A and C) or subclade or clades (Subclade A1 and Clades B and C) in the WCL neighbor joining dendrogram containing systemic isolates (Figure 5). Bootstrap values were low for both dendrograms. We did not raise bootstrap cut-off values because others have reported that gains and losses of genes may not be reflected when higher cut-off values are used in the analysis [60].

In order to estimate the discriminatory ability of the primers in the RAPD typing system and of the protein profiles, we used Simpson’s index of diversity. The Simpson’s index of diversity calculation assumes that samples are randomly selected from the population and that all groups are equally represented in the population. Samples in this study were from a few respiratory sites and mostly from diseased animals. Additionally, certain strains may be overrepresented because of their increased pathogenicity in diseased animals. However, if Simpson’s assumptions were not met, a decrease in discrimination would be expected. This was not the case in our study because differences between strains and isolates were seen in both the composite RAPD or WCP lysate results as shown in Table 3.

## Conclusions

The results of this study suggested that reference strains, “old” strains isolated in 1999, and recent field strains isolated in 2004 clustered by age of isolate when using WCL methods but not by using RAPD methods. Both the RAPD and the SDS-PAGE methods clustered strains from systemic sites. There was no strong correlation between site of isolation and genotype or between the RAPD and WCL techniques in this study. The RAPD technique showed the high heterogeneity of the *H. parasuis* isolates, whereas the protein profiles indicated that the number of passages *in vitro* of an isolate may affect its protein expression. The protein profiles of *H. parasuis* and *A. pleuropneumoniae* were unique and this WCP lysate technique may be useful as a tool to differentiate the two NAD-dependent swine respiratory organisms. The protein studies suggested that expressed genes of the organism may help to elucidate the virulence factors involved in the infection. Moreover, the relatively low cost, including supplies and equipment and relatively short amount of time required to perform the RAPD and WCP lysate methods are more advantageous when compared to other genomic or protein methods.

## Methods

### Strains and growth conditions

Fifteen *H. parasuis* type strains (reference strains A-O) were obtained from Richard Ross of the College of Veterinary Medicine, Iowa State University, Ames, Iowa and isolated between 1978 and 1990 [5,29]. Table 1 and Table 2 describe the *H. parasuis* strains used in this study. Field strains 1–24, the most recently procured in 2004, were from Lorraine Hoffman of the Veterinary Diagnostic Laboratory, Iowa State University, Ames, Iowa. Field strains 25–29 obtained in 1999 were from Karen Post, Rollins Diagnostic Laboratory in North Carolina, while field strains 30 and 31 were obtained from Richard Ross in 1999 and were originally isolated in 1984. Duplicate cultures of *H. parasuis* IA84-29755 (field strain 31), a systemic 1984 field isolate, were included in the procedures as controls.

Because of commercial unavailability of typing sera, partial serotyping with antisera to serotypes 2, 4, 5, 12, 13, and 14 of all 31 field strains was performed by Gallant Custom Laboratories, Cambridge, Ontario. Strains that did not type by agar gel immunodiffusion to the previously mentioned six serotypes were designated as “Unk” which included NT and possible other serotypes of minor prevalence in the United States and Canada. Strains were grown in Frey’s mycoplasma base broth (Sigma, St. Louis, MO) containing 20% heat-inactivated horse serum (Invitrogen, Carlsbad, CA) and 0.016% β-nicotinamide adenine dinucleotide (β-NAD) (Sigma) at 37°C overnight. Strains were checked for purity on blood agar with a nurse streak of *S. aureus* across a lawn of the *H. parasuis* isolate and on Casman’s agar (Difco, Detroit, MI) containing 5% horse serum and 0.016% β-NAD. Cultures were incubated at 37°C under humidified 5% CO_2_.

### Outgroup analysis

Strains were also studied in both RAPD and WCP lysate experiments in order to include related organisms to *H. parasuis*, of the *Pasteurellaceae* family, but ones that were not of the same species. The outgroup members serve as a reference group for determination of the evolutionary relationship among all the members of the comparison. An outgroup is hypothesized to branch from the ancestral group before the other groups branched from each other in the phylogenetic tree [61]. Selected outgroup organisms were *Actinobacillus pleuropneumoniae* (ATCC 27088), *Pasteurella multocida* (ATCC 15742), *Mannheimia haemolytica* (ATCC 43270, serotype A1), *Pasteurella trehalosi* (ATCC 29703, serotype T3), which were all members of the family *Pasteurellaceae*.

### RAPD analysis

After screening several arbitrary 10mer primers from kit A (Operon Technologies, Alameda, CA), three primers with sequences of 5’-TGCCGAGCTG-3’ (primer 2); 5’-GAAACGGGTG-3’ (primer 7); and 5’-TCGGCGATAG-3’ (primer 12) were each used individually. Primers were reconstituted in Tris-EDTA (pH 7.4) and titrated in initial assays in order to obtain the optimum amplification product. *H. parasuis* isolates, grown from 48–72 h on Casman’s agar at 37°C under humidified 5% CO_2_, were suspended in distilled water, then serially diluted 10-fold. The cell dilution that produced approximately 3 ng/μl of amplified DNA for each strain or isolate was used in the agarose gels for the RAPD experiments. Random amplified polymorphic DNA experiments were replicated three times to ensure reproducibility of the assay. The PCR mixture contained 60 mM Tris–HCl, pH 8.5, 15 mM (NH_4_)_2_SO_4_, 2 mM MgCl_2_, 0.125 mM each of dATP, dCTP, dGTP, and dTTP, 7.5 picomoles of a single 10mer, 4 μl of cell suspension, and 0.625 units of Taq polymerase (Applied Biosystems, Foster City, CA). Controls containing no *H. parasuis* cells were also included.

Amplification of DNA was performed on a GeneAmp PCR System 9600 (Perkin Elmer, Boston, MA). Cells were lysed in a “hot start” step [62] at 94°C for 10 min, and then amplified for 45 cycles of 1 min at 94°C, 1.5 min at 36°C, and 2 min at 72°C, followed by an extension step for 10 min at 72°C, then a hold step at 4°C. PCR products were stored at −20°C, until they were analyzed on 1% agarose horizontal gels in Tris-Borate-EDTA (TBE), pH 8.3 buffer [63] and detected by ultraviolet light illumination after staining with ethidium bromide. The DNA standard was a 1 kb ladder (Invitrogen, Carlsbad, CA).

### SDS-PAGE analysis

For WCP lysates, bacterial cells grown in Frey’s broth for 22 h were pelleted by centrifugation at 675 × g for 10 min. Cells were washed in 0.1 M phosphate buffered saline (PBS), pH 7.2, containing 1 mM Pefabloc (Roche Diagnostics, Indianapolis, IN), then resuspended at a ratio of 32 mg cells per 100 μl PBS/Pefabloc. Cells were sonicated with a microprobe (Heat Systems-Ultrasonics, Farmingdale, NY) at 50% power for 60 1-second bursts to lyse them and centrifuged at 16,000 × g for 20 min to remove cell debris. Protein concentrations were determined by the Folin-Lowry method [64] with bovine serum albumin as a standard.

Protein (10 μg/well) was applied to 10-well NuPAGE precast 4-12% gradient Bis-Tris gels (Invitrogen). NuPAGE antioxidant (Invitrogen) was used in 3-(N-morpholino)-propane sulfonic acid (MOPS) running buffer (Invitrogen). The protein prestained standard was BenchMark, 10–200 kDa (Invitrogen). Running conditions were 10 mA/gel for 15 min, then 200 V for 40 min. Gels were stained in 0.1% Coomassie Brilliant Blue R250 in 50% methanol/10% acetic acid and destained in 50% methanol/10% acetic acid.

### Electrophoresis pattern analysis

Gels were photographed, scanned (Kodak Image Station, Rochester, NY) and the image was digitized (Kodak Molecular Imaging Software, New Haven, CT). RAPD and protein profiles were analyzed using Gel Compar II software (Applied Maths, Austin, TX). Bands were coded as binary data (absent = 0 or present =1), regardless of band intensity. Optimal settings for band optimization and band position tolerance levels were calculated for each primer. Primer 2 values were 2.16% for band optimization and 4.72% for band position tolerance. Similarly, primer 7 values were 1.23% and 1.06%, while primer 12 values were 0.34% and 0.72%, respectively. The optimal position tolerance value gives the highest group contrast: selected scores are as high as possible within groups and as low as possible between groups. Since a band matching algorithm (Dice) was used, both tolerance and optimization were calculated. Similarity matrices were obtained from single RAPD experiments and SDS-PAGE data using the Dice similarity coefficient: *F* = 2*n*_*xy*_/(*n*_*x*_ + *n*_*y*_), where *n*_*x*_ is the total number of fragments from isolate *X*, *n*_*y*_ is the total number of fragments from isolate *Y*, and *n*_*xy*_ is the number of fragments shared by the two isolates [65]. Additionally, a combined RAPD dendrogram analysis of all three RAPD fingerprints was derived from a composite data set of the individual experiments. Neighbor joining (NJ) dendrograms were constructed with 1000 bootstrap values. Arbitrary subdivision, clades and subclades, were derived for RAPD and WCP lysate SDS-PAGE dendrograms by examining the clades as a function of percent similarity.

### Statistical analysis

Dendrograms of each single primer, composite RAPD, WCP lysate, and composite RAPD-WCP lysate were analyzed by the method of Hunter and Gaston which determines Simpson’s index of diversity D [66]. This method determines the probability that two unrelated strains from a population will be placed into different typing groups. A D-value greater than or equal to 0.9 has been determined to be necessary for confidence in typing results [66].

## Abbreviations

ATCC: American Type Culture Collection; bp: base pairs; β-NAD: β-nicotinamide adenine dinucleotide; EDTA: Ethylenediaminetetraacetic acid; ERIC-PCR: enterobacterial repetitive intergenic concensus-polymerase chain reaction; kDa: kilodalton; MEE: multilocus enzyme electrophoresis; MOPS: 3-(N-morpholino)-propane sulfonic acid; MSLT: multilocus sequence typing; NT: nontypeable; OMP: outer membrane protein; PCR: polymerase chain reaction; PCR-RFLP: polymerase chain reaction-restriction fragment length polymorphism; RAPD: random amplified polymorphic DNA; SDS-PAGE: sodium dodecyl sulfate polyacrylamide gel electrophoresis; WCP: whole cell protein.

## Competing interests

The authors declare they have no competing interests.

## Authors’ contributions

ESZ did the RAPD and WCP lysate experiments and analyzed the bands using Gel Compar II, DVL suggested the use of outgroups and provided expertise in analyzing the results, and LBT was involved in drafting the manuscript and revising it critically and served as PhD mentor for ESZ. All authors read and approved the final manuscript.
